# An Economic Evaluation of Preclinical Testing Strategies Compared to the Compulsory Scrapie Flock Scheme in the Control of Classical Scrapie

**DOI:** 10.1371/journal.pone.0032884

**Published:** 2012-03-07

**Authors:** Lisa Boden, Ian Handel, Neil Hawkins, Fiona Houston, Helen Fryer, Rowland Kao

**Affiliations:** 1 Institute of Biodiversity, Animal Health and Comparative Medicine, College of Medical, Veterinary and Life Sciences, University of Glasgow, Glasgow, United Kingdom; 2 The Roslin Institute and Royal (Dick) School of Veterinary Studies, University of Edinburgh, Midlothian, United Kingdom; 3 Oxford Outcomes, Oxford, United Kingdom; 4 Department of Zoology, The Institute for Emerging Infections, The Oxford Martin School, Oxford University, United Kingdom; Massey University, New Zealand

## Abstract

Cost-benefit is rarely combined with nonlinear dynamic models when evaluating control options for infectious diseases. The current strategy for scrapie in Great Britain requires that all genetically susceptible livestock in affected flocks be culled (Compulsory Scrapie Flock Scheme or CSFS). However, this results in the removal of many healthy sheep, and a recently developed pre-clinical test for scrapie now offers a strategy based on disease detection. We explore the flock level cost-effectiveness of scrapie control using a deterministic transmission model and industry estimates of costs associated with genotype testing, pre-clinical tests and the value of a sheep culled. Benefit was measured in terms of the reduction in the number of infected sheep sold on, compared to a baseline strategy of doing nothing, using Incremental Cost Effectiveness analysis to compare across strategies. As market data was not available for pre-clinical testing, a threshold analysis was used to set a unit-cost giving equal costs for CSFS and multiple pre-clinical testing (MT, one test each year for three consecutive years). Assuming a 40% within-flock proportion of susceptible genotypes and a test sensitivity of 90%, a single test (ST) was cheaper but less effective than either the CSFS or MT strategies (30 infected-sales-averted over the lifetime of the average epidemic). The MT strategy was slightly less effective than the CSFS and would be a dominated strategy unless preclinical testing was cheaper than the threshold price of £6.28, but may be appropriate for flocks with particularly valuable livestock. Though the ST is not currently recommended, the proportion of susceptible genotypes in the national flock is likely to continue to decrease; this may eventually make it a cost-effective alternative to the MT or CSFS.

## Introduction

Economic evaluations are a well-accepted component of the evaluation of policies to manage chronic diseases in human populations [1]. Without them, it is difficult to make a useful contribution to decision-making on disease control policy [1]. Despite this, they are often not undertaken due to the complexity and multitude of consequences (such as animal welfare, environmental protection and food security [1]) for which there often are no readily available market estimates of financial value, especially when combined with the complexities inherent in infectious disease dynamics. Additionally, as economic evaluations in healthcare are most easily interpreted when based upon experimental studies that evaluate the effectiveness of alternative strategies [2], there may be some reluctance to accept them when they use data generated from epidemiological models where nonlinear dynamics introduce additional uncertainties into the decision-making process. However, policy or individual consumption decisions (including decisions to invest resources in collecting more evidence) still need to be made given currently available data, and economic evaluations can help make this decision-making more efficient (for example by highlighting opportunity costs)

In veterinary epidemiological research many economic evaluations are based on comparisons using cost analysis (only examining costs of options) or cost minimisation (examining costs of options assuming equivalent benefits) techniques [2]. Some veterinary studies utilise true cost-effectiveness (measuring both costs and benefits) or cost-benefit analyses (measure of costs and non-equivalent effects where benefits are measured in monetary units) [3]–[6]. Few veterinary studies incorporate cost-utility analyses (benefits are measured QALYs or DALYs or other utility scale) such as found in the medical literature [7]. This study is a cost-effectiveness analysis which examines the costs and the benefits of competing scrapie control strategies considered from a societal perspective.

One example where these issues are particularly pertinent is the control of scrapie in sheep and goats. Scrapie is a transmissible spongiform encephalopathy (TSE) which results in an invariably fatal, progressive neurodegenerative disease of sheep, goats and moufflon. It is associated with an abnormal form of the prion protein (PrPSc) [8]. Other distinct transmissible spongiform encephalopathies (TSEs) have been recognized as occurring separately in humans and animals including bovine spongiform encephalopathy (BSE) (first recognized in 1986) and a new variant of Creutzfeldt-Jakob Disease (CJD) (1996). A possible link between bovine spongiform encephalopathy (BSE) in cattle and variant Creutzfeldt-Jakob Disease (vCJD) in humans [9]–[12] has resulted in an increased prioritisation of scrapie eradication in the EU and thus Great Britain (GB).

In 2001, this was acted upon in Great Britain (GB) via the National Scrapie Plan (NSP) [13], [14].The NSP's primary objectives were to eradicate scrapie and breed for TSE resistance in the national sheep flock [15], thereby minimizing the likelihood that BSE could be present and not detected in the national flock and diminishing the incidence of scrapie in the process [16]. At the time of the NSP's inception, there were no cheap or effective pre-clinical diagnostic tests available and there was speculation that the possibly low incidence of BSE in sheep might have been masked by the presence of scrapie. As a result, a genetically-based breeding strategy targeting susceptibility, rather than disease, was thought to provide the most reasonable chance for success [16]. In 2004, the NSP was augmented by a slaughter and replacement scheme. Initially, this was a voluntary programme, but after July 2004, control became mandatory for all flocks with confirmed cases from that date (Compulsory Scrapie Flock Scheme (CSFS)), as required by EC Regulations [17]–[19]. Although the CSFS is undoubtedly effective in scrapie eradication, it is not applied uniformly to all flocks within GB, as testing the national flock would be prohibitively expensive and large numbers of healthy sheep would be culled.

The development of a live test for scrapie [20] suggests that pre-clinical testing may now provide a more cost-effective disease-based strategy for scrapie eradication in the UK. The efficacy of adopting a strategy aimed at controlling disease by targeting infected sheep rather than targeting sheep at risk of being infected (due to the susceptibility of their genotype or being from an affected flock) was explored by Boden and colleagues [16]. In that study, a deterministic within-flock model was used to demonstrate that only large flocks with a large proportion of homebred breeding sheep are likely to be a significant risk for onward flock-to-flock transmission of scrapie. For most flocks it was found that the CSFS could be replaced by a strategy using a currently available live test without excessive risk to other farmer's stock, even if the proportion of susceptible genotypes in the flock is unusually large. Even for flocks that represent a high risk of harbouring a high prevalence of infection, there would be limited probability of onward transmission if scrapie is detected soon after disease introduction (typically less than 5 years). However, if detection of disease is delayed, onward transmission remains a concern and it may be more appropriate to retain the existing CSFS strategy in these flocks.

In this study we compare the direct costs and the effects of different pre-clinical testing strategies for classical scrapie. Although previous studies have considered scrapie control at the flock [11], [21]–[24] and national flock level [14], [25], [26], few have examined the economics of scrapie control policies [27], [28] and no studies have explicitly looked at the cost effectiveness of implementing such policies.

## Materials and Methods

### Model of within-flock scrapie transmission

*Within-flock scrapie prevalence after implementation of the CSFS and pre-clinical diagnostic testing strategies*

A difference equation model was used to describe the within-flock spread of scrapie for three different classifications of sheep flocks within the UK [16], [23]:

- High risk flocks (large purebred and commercial flocks (≥500 sheep) with large proportions of homebred sheep (≥0.89)
- Medium risk flocks (large commercial flocks (≥500 sheep) with small proportions of homebred sheep (≤0.10)).
- Low risk flocks (small purebred and commercial flocks (≤200 sheep) with large (≥0.89) and small proportions (≤0.10) of homebred sheep.

Lambing management and infected placental material are believed to play a large part in the transmission of scrapie, therefore flock classifications were based on the risk that scrapie affected flocks posed to other flocks through the onward sale of breeding sheep [16]. A full description of the model variables, parameters and equations is presented in Fryer *et al.* 2007 [23], reproduced in Boden *et al.* 2010 [16].

The model was adapted to consider the impact of three proposed control and eradication strategies for scrapie and contrasted with a strategy where no intervention occurs for up to 15 years, (the estimated average duration of a within-flock epidemic) [29].

There were four strategies compared:

- No intervention.
- The current Compulsory Scrapie Flock Scheme (CSFS)
- Multiple pre-clinical diagnostic tests (MT) - once a year for three years
- Single pre-clinical diagnostic test (ST) - once at a single time within a single year.

After implementation of the CSFS and the pre-clinical diagnostic testing strategies, two years of restrictions are imposed and genetically resistant replacement sheep were bought in. These strategies are described in further detail in Boden *et al.* (2010) [16].

**Figure 1:**
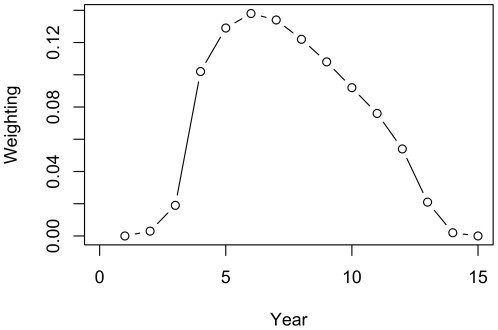
The assumed distribution of probability of detection in each year of a 15 year scrapie epidemic [30].

For each flock type, the average prevalence of scrapie per year was calculated, for every year past the initial intervention. The average prevalence was converted into number of infected sheep sold on to other farms by multiplying the prevalence and the number of infected sheep sold by each flock type. The prevalence and number of infected sheep sold in each year were summarized into a single point estimate for each flock by assuming a probability of detection in each year of a 15 year epidemic (Figure 1). This assumed that scrapie detection increased 3 years after initial infection and peaked between 5 and 7 years after initial infection. Lower probabilities of detection were assigned as the number of years since infection increased as it was assumed that the majority of cases of clinical scrapie would be detected between 5–7 years [30]. For each flock type, a distribution of flock size was obtained from the 2002 scrapie postal survey [31]. This was used to calculate testing and compensation costs for each of the control strategies in each of the flock types.

### Economic analysis

#### Cost effectiveness analysis

In the cost-effectiveness analysis, costs and the effects of each strategy were compared for each flock type (high, moderate and low risk flocks). The effects of each strategy were based on the reduction in the onward sale of infected potential breeding sheep relative to the number sold with no testing strategy.

The summary measure of cost-effectiveness was the incremental cost per infected sheep sale avoided. The use of this measure implies that societal benefit is a linear function of the number of infected sheep sold. As only a small proportion of onwards sales will be to breeding stock we have assumed that the onwards risk of transmission and hence societal impact is linearly related to the number of infected sheep sold.

Alternative control strategies were considered according to their effectiveness and their costs. Any strategies where there was an alternative that was both cheaper and more effective were removed from the comparison as these are ‘dominated’ strategies [2]. Strategies subject to extended dominance [2], where an alternate strategy can be replace by a more cost effective mix of alternatives, were not removed as we made the assumption that strategies may have to be applied to either all flocks nationally, or all flocks in a risk stratum.

### Costs

The sum of the total costs for travel, time (veterinarian and helper), sampling consumables, testing, examination, and reporting for genotype testing and costs of compensating the farmer for sheep removed from the flock were calculated for each strategy for each year since the flock was infected (in a 15 year epidemic). A description of these costs is outlined in Table 1. As market-based estimates of the cost of the pre-clinical test were not available, a threshold analysis was performed (using the base scenario with a test sensitivity of 90% and prevalence of 40% susceptible genotypes in the high risk flocks). The pre-clinical test was initially assumed to have the same unit cost and volume discounting costs of the genotyping. This unit cost was then multiplicatively scaled to identify the threshold at which the total cost of the multiple testing strategy was equal to the total cost of the CSFS.

**Table 1:** Description of costs incurred for Compulsory Scrapie Flock Scheme, single and multiple pre-clinical testing strategies.

Costs	Description
Compensation for the compulsory scrapie flock scheme (CSFS) and diagnostic testing strategies	The mean number of sheep that the farmer is compensated for is equal to the proportion of susceptible sheep * flock size.Where:a.Proportion of genetically susceptible sheep = 0.40b.Proportion of ewes in flock = 0.98c.Proportion of rams in flock = 0.02d.Cost per ewe £65e.Cost per ram £90This cost is constant over each year of an epidemic (i.e. cost of compensation does not depend on prevalence of scrapie in flock). We assumed that the proportion of susceptible sheep in each flock will be reflected equally across the proportions of ewes and rams.
Compensation for multiple and single testing strategies	Compensation is paid for each sheep positive for scrapie as detected by the pre-clinical test. The test is assumed to have 90% sensitivity and 100% specificity. Therefore, the mean number of sheep detected as positive for scrapie is dependent on the prevalence of scrapie in the flock in each year since the flock was infected multiplied by 0.90. In the multiple testing strategy, the entire flock is tested each year for three years and the farmer is compensated for the total mean number of sheep detected as positive in years 1, 2 and 3. This total number is never greater than the total number of infected sheep in the year since infection because it is assumed that the prevalence does not substantially increase in the second and third year of testing (e.g. mean number of sheep detected in year 2 = 0.9* (number of infected sheep in year 1−number of infected sheep detected and removed by the test in year 1).Proportion of ewes positive for scrapie = 0.98*prevalenceProportion of rams positive for scrapie = 0.02*prevalenceCosts per ewe £65Cost per ram £90
Travel (All strategies	From base to farm; 40 pence/mile @ 45 miles/h. We assumed that the average farm was half an hour away from base.
Time (All strategies)	Veterinarian = £70/hour; helper = £40/hour; @ 40 samples/hour
Sampling consumables(All strategies)	Speculum, forceps, scissors, containers = £5/sample
Genotype testing(CSFS only)	*1–10 samples @ £ 29.00 per sample;*11–29 samples @ £22.50 per sample;*Subsequent numbers of sheep test @ £14.50 per sampleFarmer can do the sampling themselves if sheep are not for export.If sheep are for export, then additional veterinarian/helper charges are also considered in this cost.
Examination of preclinical test samples(Testing strategies only)	Market based price data not available. The pre-clinical test was initially assumed to have the same unit cost and volume discounting costs of the genotyping. This cost was then multiplicatively scaled to identify the threshold at which the total cost of the MT strategy was equivalent to the total cost of the CSFS strategy in the 90% sensitivity and 40% prevalence scenario. (i.e. the point at which the MT strategy becomes dominated by CSFS).

The ‘average’ unit cost of pre-clinical testing at the threshold price was calculated by dividing the sum of the cost of testing all flocks by total number of sheep tested. Cost Effectiveness analysis was subsequently used to compare the strategies where applicable. Sensitivity of the results to preclinical test cost was explored by repeating the analysis with the preclinical test unit cost set at 0.5 and 1.5 times the threshold value.

### Assumptions

We assumed that the cost and effort of replacing stock (culled as a consequence of removal from the flock due to scrapie) in the two years following each of the three strategies was equivalent to the current level of compensation offered by Defra. The costs of scrapie testing (genotype or pre-clinical testing) and compensation were assumed to be constant over each year of an epidemic (i.e. cost of compensation did not depend on prevalence of scrapie in flock). We assumed that the proportion of susceptible or diseased sheep in each flock would be distributed equally across the proportions of ewes and rams in eafch flock.

The additional value of pedigree and pregnant sheep were not factored into these analyses. The calculation of the costs of the testing strategies did not take into account the costs for shipment of samples from base to the laboratory where samples were examined. Additionally these costs did not take into account the costs for the time of the farmer/stockmen to help handling the samples.

### Consequences

#### Reduction in the number of infected sheep sold

The number of infected sheep sold by each flock was calculated from the deterministic model [16], [23] parameterised with data from the scrapie postal survey [31]. The number of infected sheep sold for each control strategy was then subtracted from the number of infected sheep sold would there to be no control strategy to give the reduction in the number of infected sheep sold to other farms. The number of infected sheep sold after implementation of the CSFS is always assumed to be zero as this strategy removes all susceptible genotypes within the flock.

### Sensitivity analyses

The base analysis assumed a pre-clinical test sensitivity of 90% and that 40% of each flock had a scrapie susceptible genotype. The within-flock model was re-run and economic analyses were repeated to allow for differences in the test sensitivity (70%, 50%) and proportions of susceptible genotypes in the flock (30%, 20%,10%).

The model was initially implemented in excel and then independently written in R statistical software to validate the results.

## Results

**Figure 2:**
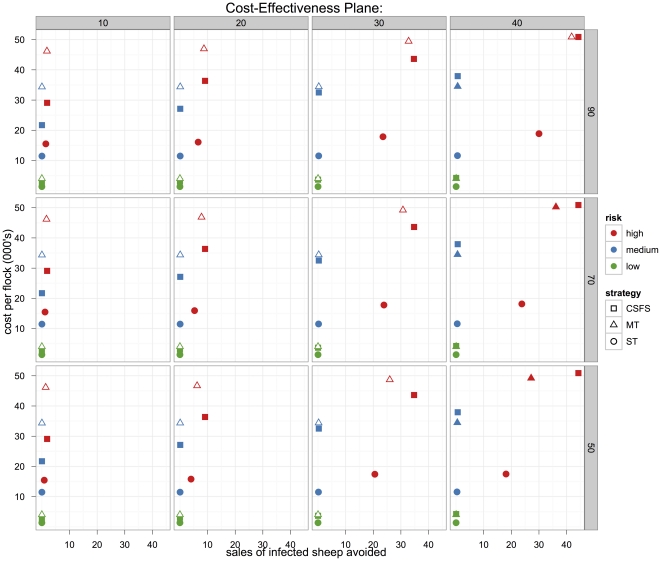
Cost-effectiveness plane examining all flocks (high, moderate and low risk) with different proportions of susceptible genotypes. In this analysis, we have found the unit cost of pre-clinical testing that makes the total CSFS and MT (including compensation, labour and testing) equivalent costs. The cost effectiveness planes vary by test sensitivity (rows 50–90%) and proportions of susceptible genotype (columns 10–40%) The outlined points on the plot represent dominated strategies. The origin in each panel represents no intervention. In high-risk flocks, the CSFS and MT are more efficient than the single test strategy (ST) at high proportions of susceptible genotypes within the flock (i.e. >30%). At lower proportions of susceptible genotypes (i.e. ≤30%) in high risk flocks, MT is the dominated strategy. MT has to be cheaper, and this is exacerbated if prevalence or test sensitivity drops.

The effectiveness of a strategy, as measured by avoiding the onwards sale of infected sheep compared to no control strategy, is a function of the strategy, the flock risk class and the proportion of susceptible sheep within the flock (and test sensitivity for the pre-clinical testing based strategies). Based on the base costings, the contribution of compensation costs for culled animals was 57% for CSFS, 19% for MT and 10% for ST. Cost and effectiveness results at the threshold cost for preclinical testing are shown in Figure 2. For each sensitivity analysis, scenario ST was the cheapest strategy at the threshold unit cost for preclinical testing. Likewise ST was least effective and CSFS most effective. For all but the high risk flocks only low (<0.7) numbers of infected sheep sales were avoided. The resulting incremental costs effectiveness ratios in the low and medium risk flocks were relatively high ranging from £12,000 per sale avoided to £2.6 M per sale avoided, with the upper end representing the high cost of attempting control in effectively resistant flocks (*R_0_* below one). Unless the value of avoiding sales of infected sheep exceeds £12,000 none of the three testing strategies would be considered cost-effective in the low and medium risk flocks. The following description and discussion of the results will focus on the high-risk flocks.

To achieve equivalent overall costs for MT and CSFS in the high-risk flocks, assuming within-flock prevalence of 40% susceptible genotypes and a test sensitivity of 90% the ‘average’ unit cost of pre-clinical testing had to be set at £6.28.

**Figure 3:**
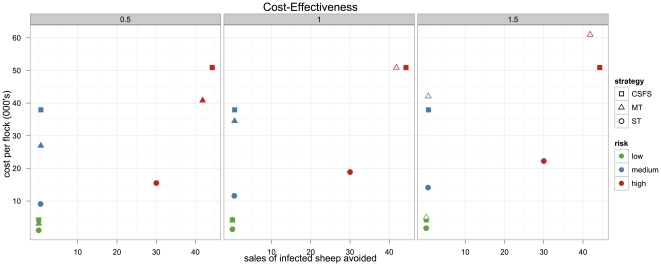
Cost-effectiveness plane examining all flocks (high, moderate and low risk) assuming 40% proportion of susceptible genotypes under three preclinical test unit costs (0.5, 1.0 and 1.5 times threshold price that would give CSFS and MT equal costs). The MT strategy is clearly dominated in the 1.5 times scenario having a much greater cost than CSFS with lower effectiveness (fewer infected sheep sales avoided.).

Costs, effects and incremental cost effectiveness ratios (ICER – the incremental cost of avoiding the sale of an additional infected sheep) are presented in Table 2 for the base scenario with a test sensitivity of 90% when the proportion of susceptible genotypes in the flock is 40%. The cost/effectiveness of each testing strategy is shown in Figure 3 for the threshold cost of pre-clinical testing and scenarios where pre-clinical testing is 0.5 and 1.5 times its threshold cost.

**Table 2:** Costs and effects (infected-sales-averted) for each scrapie control strategy assuming Defra pay all costs.

Preclin test cost	Strategy	Mean Infected Sales Per Flock	Incrmntal Infected Sales Avoided	Mean Number of Sheep Tested	Mean number of sheep slaughtered	Cost of Sheep Slaughtered	Cost of Testing	Total Cost	IncrmntalCost (GBP)	IncrmntalEffect	ICER (cost per infected sale avoided)
base	None	44.26	0	0	0	0	0	0			-
(£6.28 unit cost)	ST	14.25	30.01	1109	54	3535	15335	18870	18870	30.01	629
	MT	2.44	41.82	3327	75	4885	46006	50891	32021	11.81	dominated
	CSFS	0	44.26	1109	444	29068	21823	50891	32021	14.25	2247
preclin testing ×1.5	None	44.26	0	0	0	0	0	0			-
(£9.42 unit cost)	ST	14.25	30.01	1109	54	3535	18698	22233	22233	30.01	741
	MT	2.44	41.82	3327	75	4885	56098	60983	38750	11.81	dominated
	CSFS	0	44.26	1109	444	29068	21823	50891	28658	14.25	2011
preclin testing ×0.5	None	44.26	0	0	0	0	0	0			-
(£3.14 unit cost)	ST	14.25	30.01	1109	54	3535	11971	15506	15506	30.01	517
	MT	2.44	41.82	3327	75	4885	35914	40799	25293	11.81	2142
	CSFS	0	44.26	1109	444	29068	21823	50891	10092	2.44	4136

This illustrates the costs and effects for high risk farms using CSFS and pre-clinical tests with a sensitivity of 90%, when the proportion of genotypes in the flock is 40%. The incremental cost effectiveness ratio (ICER) is also presented where appropriate.

In the high-risk flocks, assuming within-flock prevalence of 40% susceptible genotypes and a test sensitivity of 90%, the ST was the cheapest (£18,870) but least effective of the three testing strategies (30 infected-sales-averted). The MT (42 infected-sales-averted) was slightly less effective than the CSFS (44, i.e. all, infected-sales-averted). MT/CSFS costs for testing and compensation were £50,891.

Compared to no-intervention the ST strategy reduces infected sheep sales at £629 per sale averted (14.25 infected sheep sold compared to 44.26). CSFS reduces infected sheep sales to zero at an incremental cost per sale averted (ICER) of £2247.

If the average unit-cost per sheep of pre-clinical testing was scaled upwards the MT strategy was dominated, with the CSFS strategy costing less and reducing infected sheep sales to zero. With a preclinical test unit cost of 1.5 times the threshold cost (i.e. £9.41) the ST strategy reduced sales of infected sheep at £741 per sale averted compared to no intervention and CSFS had an ICER compared to ST of £741.

If the average unit-cost per sheep of pre-clinical testing was scaled downwards (0.5 times the equivalent unit-cost of preclinical testing) no strategies were dominated with ST having a cost per infected sale avoided of £517, MT compared to ST of £2142 and CSFS compared to MT of £4136.

In high risk flocks, decreasing the sensitivity of the diagnostic test reduced the efficacy of the multiple (MT) and single (ST) test strategies. At lower proportions of susceptible genotypes (i.e. ≤30%), MT is the dominated strategy. Thus as test sensitivity and proportions of susceptible genotypes decreases, the cost of MT has to be increasingly less than the threshold cost for it to be considered a reasonable alternative to the CSFS. If the prevalence of susceptible genotypes decreases over time, the difference in the effectiveness between the ST and MT/CSFS will become smaller. Under those conditions, in high risk flocks, the ST could be an alternative to the MT or CSFS.

## Discussion

This study compares the costs and effects of adopting a diagnostic testing strategy (either single or multiple testing) instead of the current CSFS to diagnose and eradicate classical scrapie in GB sheep flocks. These analyses are only applicable for flocks that have been identified as having at least one clinical case of scrapie, which would result in the mandatory application of a scrapie control measure (CSFS or one of the testing strategies).

The efficacy of each of the alternate strategies compared to the CSFS was examined in Boden *et al.* (2010) [16] and provided the data for the efficacy measures used in this study. In that study, Boden *et al.* (2010) [16] recommended that for most flocks (moderate and low risk) the CSFS could be replaced by a strategy using a currently available live test without excessive risk to other farmers, even if the proportion of susceptible genotypes in the flock is unusually large. For high risk flocks, it may be more appropriate to retain the existing CSFS strategy in these flocks [16].

In this study, it is assumed that with the CSFS, zero infected animals will be sold. However, based on genotype, some sheep are more resistant than others and it is possible that a resistant animal can be infected with scrapie, especially if it is not ARR/ARR. As such, it is possible that scrapie in non-ARR/ARR sheep may be retained in the CSFS strategy and some onward movement of infection between flocks may occur. However, it is expected that in the current environment, the within-flock R0 for most flocks will remain low. Additionally, the long incubation periods associated with more resistant sheep suggest that their contribution to new outbreaks is small and unlikely to have a major impact on the results of this study.

This study does not address any costs associated with atypical scrapie. Even though breeding for resistance to classical scrapie does not select for resistance to atypical scrapie, selection of ARR homozygote genotypes is unlikely to increase the prevalence of atypical scrapie [32] and to date, there has been no evidence that atypical scrapie is either transmissible or a potential zoonosis [33]–[35]. Therefore selection towards resistance to classical scrapie is unlikely to have meaningful economic consequences, even if it does result in a small increase atypical scrapie prevalence.

The results of this economic analysis support the replacement of the CSFS with a single testing strategy in low and moderate risk flocks *if* a control strategy is mandatory and the testing and compensation costs are borne by Defra; in these instances, a single pre-clinical diagnostic test strategy is the cheapest strategy compared to the MT and the CSFS.

In high risk flocks, at high proportions of susceptible genotypes and high test sensitivity, CSFS strategies would be recommended if the additional benefits of avoiding the onwards sale of approximately 14 sheep obtained by CSFS were worth at least the estimated £2247 per sale averted.

The CSFS strategy results in zero onwards infected sheep sales (2.44 less than the MT strategy) however if the unit cost of preclinical testing were sufficiently low the additional cost of avoiding these sales may be judged unacceptable and MT may be the preferred strategy. For example if the preclinical test unit cost was £3.14 per sheep, on average, the reduction in onwards infected sheep sales from 2.44 to zero would cost £4136 per sale averted. A move from CSFS raises the issue of disinvestment. In economic evaluation this is an apparent asymmetry of decision making whereby interventions are adopted at a lower cost per unit of benefit than they are abandoned. It may be that moving to a strategy which is only slightly less effective (MT) may be considered unacceptable even it markedly reduces costs.

If the preclinical testing unit cost was greater than or equal to the threshold value (£6.28 per test) MT would be dominated and not a rational option. When the unit cost of pre-clinical testing is only slightly lower then the threshold value MT is subject to extended dominance [36]. This implies that even though it is less costly than CSFS a more cost effective strategy could be obtained by applying a mix of ST and CSFS which would have a lower cost than MT for the same, average, number of infected sales averted. This would require a mixed strategy to be technically and politically feasible and makes the strong assumption that the effectiveness scale of sales avoided maps linearly to societal benefits. In other words, in terms of scrapie control and eradication strategies, a mixed strategy using a combination of genotype and pre-clinical testing in flocks may under certain test unit cost conditions be the most cost-effective option. Although this may be the most efficient allocation of resources, it may not be the most fair or equitable [36]. Different sheep would be exposed to different control measures (and outcomes) within flocks. We have not considered sensitivity to the costs of genotyping as these are well-established. However, should these change, for example if a reduction in the volume of samples genotyped results in a concomitant increase of per unit cost, this would potentially increase the relative attractiveness of a pre-clinical test based strategy. Similarly, we base this analysis on an average compensation rate for sheep – some pedigree breeding stock are dramatically more valuable than others, and therefore may result in very different decision points if flocks are considered on an individual basis. As losses to a farmer may exceed compensation costs in the case of higher valued pedigree animals, the relative costs of different strategies may vary. Specifically, the cost of the CSFS strategy is highly influenced by compensation costs and may make MT a potentially desirable strategy in high value flocks.

If there are no budget constraints, the CSFS would achieve the most effective outcome and would be the most fairly and ethically applied strategy across the population [36].

Of course the sensitivity of the pre-clinical test and the proportion of susceptible genotypes in the flock will alter the cost-effectiveness of different control strategies in high risk flocks. As test sensitivity and proportions of susceptible genotypes decreases, the cost of MT has to be increasingly cheaper than current estimates for it to be considered a reasonable alternative to the CSFS. If the prevalence of susceptible genotypes decreases over time [37] as has also been seen in the Netherlands [38], in high risk flocks the difference in the effectiveness between the ST and MT/CSFS will become smaller. Under those conditions, the ST could become an alternative to the MT or CSFS in the future.

At present, this analysis was simplified to only consider the costs and effects of each strategy in the present (i.e. no discounting of costs and effects was applied). A deterministic sensitivity analysis was used to examine the impact of test sensitivity and proportion of susceptible genotypes in the flock.

We have compared the efficacy of each strategy by comparing the reduction in the number of infected sheep sold. However, we recognize that there is potentially more than one measure of effect for each strategy for each scenario. For example, from a government/industry perspective, the risk of disease spread and subsequent trade restrictions and animal welfare may be the most important effects to measure the efficacy and ultimately effectiveness of a successful control strategy. Alternatively, a farmer may consider loss of performance traits, the effect of inbreeding, loss of genetic diversity, susceptibility to other diseases and trade restrictions more important. Equally, a societal perspective may also take into account consumer interest in disease-free meat. Measuring multiple effects that may occur during a scrapie epidemic (such as trade and food security disruption and animal welfare) and measured in the context of economic analyses may be best represented by a cost-benefit analysis [2] and this will be considered in future studies.

In this study, we have shown that extending a previous epidemiological analysis [16] to consider economics presents additional options that may have a considerable benefit to animal health and welfare. Such combined epidemiological economic analyses are in their infancy when considering infectious disease dynamics, but are often important when considering policy advice that must consider the complexities of nonlinear infectious disease dynamics.
